# Nucleobase and nucleoside transport and integration into plant metabolism

**DOI:** 10.3389/fpls.2014.00443

**Published:** 2014-09-09

**Authors:** Christopher Girke, Manuel Daumann, Sandra Niopek-Witz, Torsten Möhlmann

**Affiliations:** Pflanzenphysiologie, Fachbereich Biologie, Universität KaiserslauternKaiserslautern, Germany

**Keywords:** plant nucleotide metabolism, nucleobase transport, nucleoside transport, uridine, adenosine, uracil

## Abstract

Nucleotide metabolism is an essential process in all living organisms. Besides newly synthesized nucleotides, the recycling (salvage) of partially degraded nucleotides, i.e., nucleosides and nucleobases serves to keep the homeostasis of the nucleotide pool. Both types of metabolites are substrates of at least six families of transport proteins in *Arabidopsis thaliana* (*Arabidopsis*) with a total of 49 members. In the last years several members of such transport proteins have been analyzed allowing to present a more detailed picture of nucleoside and nucleobase transport and the physiological function of these processes. Besides functioning in nucleotide metabolism it turned out that individual members of the before named transporters exhibit the capacity to transport a wide range of different substrates including vitamins and phytohormones. The aim of this review is to summarize the current knowledge on nucleobase and nucleoside transport processes in plants and integrate this into nucleotide metabolism in general. Thereby, we will focus on those proteins which have been characterized at the biochemical level.

## Introduction

Nucleotide metabolism is an essential process in all living organisms as nucleotides function as energy providers, signals and building blocks for nucleic acids as well as the plant hormone cytokinin. Nucleotide metabolism can be structured into *de novo* synthesis, salvage and catabolism with corresponding transport processes. During *de novo* nucleotide synthesis nucleoside monophosphates are synthesized from amino acids, bicarbonate, organic acids, and phosphoribosyl pyrophosphate (PRPP). In addition, the reducing equivalents adenosine-5′triphosphate (ATP) and tetrahydrofolate (THF) are required (Zrenner et al., 2006). All organisms are capable of performing such nucleotide *de novo* synthesis. The only known exceptions are human pathogenic protists lacking purine *de novo* synthesis and compensating for this lack by import of nucleobases or nucleosides from the host cells, which are then recycled to nucleotides by the salvage pathway (Mäser et al., 1999). According to the current view purine nucleotide *de novo* synthesis is finalized in plastids and the resulting monophosphates have to be delivered to other compartments in which nucleotides are needed.

In the salvage pathway nucleobases can be converted to the corresponding monophosphates by action of phosphoribosyl transferases (PRTs). In addition nucleosides can be phosphorylated by nucleoside kinases (NKs). Both pathways are less energy consuming than *de novo* synthesis and allow preserving nitrogen and energy in nucleobases and nucleosides. Catabolism of nucleosides and nucleobases allows the liberation of nitrogen in form of ammonia to be reassimilated in the glutamine oxoglutarate aminotransferase (GOGAT) pathway (Jung et al., 2009, 2011; Zrenner et al., 2009; Cornelius et al., 2011; Werner and Witte, 2011). Especially intermediates of purine catabolism such as allantoin and allantoate might exhibit distinct physiological functions such as acting as scavengers for reactive oxygen species (ROS; Brychkova et al., 2008). In some legumes, allantoin and allantoic acid are the main products of nitrogen fixation and serves as the primary nitrogen transport form Christensen and Jochimsen (1983), Smith and Atkins (2002). Accordingly, specialized transporters (ureide permeases) are required for the distribution of ureides in these plant species.

Purine *de novo* synthesis from PRPP and glutamine (Gln) to inosine monophosphate (IMP) involves nine enzymatic steps (Zrenner et al., 2006). The first enzymatic reaction is catalyzed by the glutamine phosphoribosyl pyrophosphate amidotransferase (ATase) which is encoded by one to four homologous genes in Angiosperm species according to the Phytozome database (Goodstein et al., 2012; www.phytozome.net). In *Arabidopsis* three ATase genes are present. Knockout of ATase2 is characterized by pale green mosaic leaves, also known as reticulated leaf mutant and a 50% reduction in leaf cell size indicating the role of purine *de novo* synthesis in cell division (Hung et al., 2004; van der Graaff et al., 2004; Lundquist et al., 2014). All other steps, except for adenylosuccinate lyase which is encoded by two genes, are single copy genes in *Arabidopsis* (Zrenner et al., 2006). No homozygous mutants for any of these genes are described following the logic that no free living organism can develop without nucleotide *de novo* synthesis. However, it is not reported whether *de novo* synthesis is performed in each individual plant cell or if some cells depend on salvage pathway activity and thus on import of corresponding precursors. In early plant development it is supposed that the production of nucleotides mainly depends on purine and pyrimidine salvage rather than *de novo* synthesis which dominates at later phases (Ashihara, 1983; Ashihara et al., 1997; Stasolla et al., 2003). So far, the only known transporter mediating export of newly synthesized purine nucleotides out of plastids is the *Arabidopsis* Brittle 1 protein (Kirchberger et al., 2008).

The initial reaction in pyrimidine *de novo* synthesis is the formation of carbamoyl phosphate by carbamoyl phosphate synthase. This enzyme is composed of two subunits. Mutants in both *CarA* and *CarB* were identified in *Ven3* and *Ven6* and exhibited a reticulate phenotype, which means that the leaf parenchyma cells are chlorotic or degenerated whereas the cells around the vasculature stay green, reviewed in Lundquist et al. (2014). The four subsequently acting proteins aspartate transcarbamoylase (ATCase), dihydroorotase (DHOase), dihydroorotate dehydrogenase (DHODH) and uridine-5′-monophosphate synthase (UMPSase) are encoded by single genes in *Arabidopsis*, potato and tobacco (Giermann et al., 2002; Zrenner et al., 2006).

Antisense repression of all genes of pyrimidine *de novo* synthesis in potato and tobacco revealed that about 20% of wildtype transcript levels are sufficient for plant growth. Below this level growth restrictions become apparent. In case of DHOase and ATCase a clear correlation between protein amount and growth could be detected, pointing out the importance of pyrimidine *de novo* synthesis for plant development (Schröder et al., 2005). The subcellular distribution of *de novo* synthesis points out the necessity of the plastidic nucleobase transporter PLUTO, described in detail in the chapter “The nucleobase:cation symporter 1 family.”

When nucleosides or nucleobases are imported into a plant cell by one of the many transport proteins present in plants they can undergo two fates. (1) They can be recycled to nucleoside monophosphates by PRTs or NKs. (2) They are subjected to complete degradation by which nitrogen is liberated in form of ammonia and may then be reassimilated to synthesize amino acids. According to the results of the analysis of mutants in catabolic enzymes as well as flux measurements using radiolabeled nucleosides and nucleobases it can be concluded that nucleosides are more effectively salvaged compared to nucleobases. In other words, the hydrolysis of nucleosides to nucleobases by nucleoside hydrolases (NSHs) results in a shift toward catabolism. This allows the assumption that nucleoside import into cells mainly supports salvage whereas nucleobase import supports catabolism.

In the cell relative adenylate contents determine the cellular energy charge whereas outside ATP seems to be the primary signal (Roux and Steinebrunner, 2007; Tanaka et al., 2010; Möhlmann et al., 2013). Such extracellular ATP can result from wounding of cells, export by vesicle flow or by direct transport by PmANT1 (Plasma membrane Adenine Nucleotide Transporter 1; Rieder and Neuhaus, 2011). The sensing of this extracellular ATP is mediated by the recently discovered receptor DORN1 (Does not Respond to Nucleotides 1; Choi et al., 2014).

The removal of the ATP signal is mediated by ATP cleaveage by nucleoside triphosphate diphosphohydrolases (NDPDases) or phosphatases. Which enzymes are in charge for this process has not been clarified so far. However, a complete degradation of ATP down to the nucleobase adenine was shown to proceed in potato tuber apoplastic extracts (Riewe et al., 2008).

In the following sections the different protein families for transport of nucleosides and nucleobases will be described. All members of these families which were characterized at the molecular level including their substrates, expression pattern and physiological function are listed in Tables 1–3.

**Table 1:** **Nucleoside transporters of the ENT family**.

**Species (number of members)**	**Transporter (accession number)**	**Expression system, K_M_ [μM], substrate**	**Expression pattern**	**Physiological function**	**References**
**2.A.57.1 EQUILIBRATIVE NUCLEOSIDE TRANSPORTER (ENT)**					
*Arabidopsis thaliana* (8 members)	AtENT1 (At1g70330)	Y 3.6 (Adenosine)	Nearly constitutively expressed, high in pollen and leaf hydathodes	Export of nucleosides from the vacuole derived from RNA degradation	Li and Wang, 2000; Möhlmann et al., 2001; Li et al., 2003; Sun et al., 2005; Bernard et al., 2011
		Y 30.0 (Cytidine)			
		Y 3.9 (Uridine)			
	AtENT3 (At4g05120)	Y 15.5 (Adenosine)	Vasculature of leaves and roots	Main uridine uptake system, Nucleoside uptake for salvage and degradation (liberation of nitrogen), long distance transport	Li et al., 2003; Wormit et al., 2004; Sun et al., 2005; Chen et al., 2006; Traub et al., 2007; Möhlmann et al., 2010; Cornelius et al., 2012
		Y 10.0 (Cytidine)			
		Y 18.0 (Guanosine)			
		Y 2.3 (Thymidine)			
		Y 9.5 (Uridine)			
	AtENT4 (At4g05130)	Y 94.2 (Cytidine)	Leaves, flowers and stem	Nucleoside uptake for salvage	Li et al., 2003; Wormit et al., 2004
		Y 7.3 (Guanosine)			
		Y 27.8 (Uridine)			
	AtENT6 (At4g05110)	Y 3.0 (Adenosine)	Roots, leaves and flower vasculatures, stomata	Putatively uptake of nucleosides into cells, transport of cytokinin ribosides, long distance transport	Wormit et al., 2004; Hirose et al., 2008
		Y 21.2 (Cytidine)			
		Y 11.5 (Guanosine)			
		Y 17.0 (iPR)			
		Y 630.0 (tZR)			
		Y 6.4 (Uridine)			
	AtENT7 (At1g61630)	Y 9.8 (Adenosine)	Leaves and flowers, high in pollen		Wormit et al., 2004
		Y 40.0 (Cytidine)			
		Y 9.4 (Guanosine)			
		Y 13.4 (Uridine)			
*Solanum tuberosum*	StENT1 (FR719954)	Y 4.0 (Adenosine)	N.A.		Bernard and Möhlmann, unpublished
		Y 17.6 (Cytosine)			
		Y 30.2 (Guanosine)			
		Y 6.8 (Uridine)			
	StENT3 (FR719955)	Y 45.5 (Cytidine)	N.A.		Bernard and Möhlmann, unpublished
		Y 12.6 (Uridine)			
*Hordeum vulgare* (2 members)	HvENT1 (LK391769)	Y 14.1 (Adensosine)	N.A.		Niopek-Witz and Möhlmann, unpublished
		Y 3.6 (Cytidine)			
		Y 3.7 (Uridine)			
*Oryza sativa* (4 members)	OsENT2 (Os07g37100)	Y 3.0 (Adenosine)	Predominantely in roots	Retrieval of endosperm-derived nucleosides, long distance transport	Hirose et al., 2005
		Y 32.0 (iPR)			
		Y 660.0 (tZR)			
		Y 0.7 (Uridine)			

iPR, isopentenyl adenine riboside; N.A., not analyzed; RNA, ribonucleic acid; tZR, trans-zeatin; Y, expressed in yeast.

## The equilibrative nucleoside transporter family

Equilibrative nucleoside transporters (ENTs) represent a family of integral membrane proteins present in a wide range of eukaryotic organisms and mediating transport of hydrophilic nucleoside substrates. Substantial progress has been made in characterization of mammalian ENTs in the last decades due to their medical importance (Cabrita et al., 2002; Baldwin et al., 2003; King et al., 2006; Young et al., 2013), whereas knowledge about plant ENTs is still limited. First descriptions of plant nucleoside transporters appeared in 2000 and 2001 (Li and Wang, 2000; Möhlmann et al., 2001). In contrast to nucleobase transport, nucleoside transport in plants is mediated by members of just one transporter family: ENTs. Members from four plant species have been characterized at the biochemical level to date – AtENT1, 3, 4, 6, and 7 from *Arabidopsis*, OsENT2 from rice, HvENT1 from barley and StENT1 and 3 from potato (Table 1). In contrast to mammalian homologs which mediate an equilibrative transport of substrates along a concentration gradient, most plant ENT proteins function as substrate-proton symporters (Li et al., 2003; Wormit et al., 2004; Hirose et al., 2005; Traub et al., 2007). The only known exception so far represents AtENT7 which was shown to catalyze a nucleoside transport in yeast that was hardly affected by the protonophore carbonyl cyanide m-chlorophenyl hydrazone (CCCP) and the pH value of the medium (Wormit et al., 2004). Nucleoside transport has been studied after heterologous expression of the corresponding genes in yeast and Xenopus oocytes. All five *Arabidopsis* proteins analyzed so far (AtENT1, AtENT3, AtENT4, AtENT6, and AtENT7) exhibit broad substrate specificity and transport the purine nucleosides adenosine and guanosine, as well as the pyrimidine nucleosides cytidine and uridine. The apparent K_M_ values were in the range from 3 to 94 μ M (Table 1). Transport was strongly inhibited by deoxynucleosides and to a lesser extent by nucleobases (Möhlmann et al., 2001; Li et al., 2003; Wormit et al., 2004). Typical inhibitors of mammalian ENT proteins, such as dilazep and nitrobenzylmercaptopurine ribonucleoside (NBMPR) surprisingly exerted almost no effect on *Arabidopsis* ENT proteins.

An *AtENT3* mutant was identified as FUR1 and contains a point mutation in the *AtENT3* gene which leads to an exchange of glycine to arginine at position 281 and to the loss of function (Wu and King, 1994; Traub et al., 2007). *FUR1* mutants and *AtENT3* T-DNA mutants are completely resistant against the toxic pyrimidine nucleoside analog 5-fluorouridine (5-FU). This lead to the identification of AtENT3 as the major pyrimidine importer in *Arabidopsis* seedlings (Chen et al., 2006; Traub et al., 2007). Since NSH3—an extracellular purine specific nucleoside hydrolase—cleaves inosine and adenosine purines can still be imported in form of nucleobases in *AtENT3* mutants (Jung et al., 2011). Gene expression of all six AtENTs could be observed during seed germination and development. Whereas the transcript level of AtENT1 remained relatively constant, the transcript of AtENT3, 4, 6, 7, and 8 steadily increased while germination proceeded (Chen et al., 2006). Expression analysis with 10 day old seedlings indicated relatively high transcript levels for AtENT1 and AtENT3 whereas the transcript of all other AtENTs was markedly lower (Cornelius et al., 2012). Furthermore, expressional analysis of 7 week old plants revealed the presence of AtENT1 transcript in root cells, stem, flower and siliques (Li et al., 2003). Promotor GUS stainings supported these findings and indicated high AtENT1 transcript additionally in pollen (Bernard et al., 2011).

Although AtENT1 and AtENT3 are expressed in root cells, only *AtENT3* T-DNA mutants showed a reduced uptake of uridine or the cytotoxic uridine analog 5-FU (Li et al., 2003; Traub et al., 2007; Cornelius et al., 2012). The presence of AtENT1 in two independent tonoplast proteome analysis (Jaquinod et al., 2007; Schulze et al., 2012) undermined the belief that AtENT1 is present at the plasma membrane (Li and Wang, 2000). Correlation between overexpression of AtENT1 and lower adenosine and 2′:3′ adenosine monophosphate (2′:3′-cAMP) contents in the vacuole, which appear as breakdown products of vacuolar RNA degradation gave hints for a participation of AtENT1 in transport processes of RNA break down products. As a proton symporter AtENT1 exports nucleosides from the vacuole, leading to the question about the physiological function of such a vacuolar nucleoside transport activity. It has been reported that vacuoles from tomato cell culture contain nucleosides and further RNA breakdown products (Leinhos et al., 1986; Abel et al., 1990). Therefore, one might speculate that RNA degradation partially takes place in the vacuole and AtENT1 is involved in export of liberated nucleosides for salvage or catabolism. Vacuolar RNase isoforms were identified in tomato and *Arabidopsis* (RNS2; Abel and Glund, 1987; Löffler et al., 1992). Beyond, RNS2 mutants exhibited reduced RNA degradation activity (Hillwig et al., 2011). The same authors provided evidence for an entry of RNA into vacuoles by autophagy.

Four genes coding for potential equilibrative nucleoside transporters were identified in rice, designated OsENT1-4. Analysis in yeast cells expressing OsENT2 revealed high affinity purine and pyrimidine transport (Hirose et al., 2005). Interestingly transport was also affected in the presence of several deoxy-nucleosides and in addition cytokinin type nucleosides (isopentenyl adenine riboside, iPR; *trans*-zeatin riboside, tZR) were transported (Hirose et al., 2005). Therefore, OsENT2 represents a nucleoside transporter in rice with a broad substrate spectrum. In addition, direct evidence for a participation of *Arabidopsis* ENT6 in cytokinin nucleoside transport with a preference for iPR was gained (Hirose et al., 2008). Furthermore, Sun et al. (2005) were able to provide indications for an involvement of AtENT3 and AtENT8 in cytokinin transport. However, whether these data reflect a participation of ENT proteins in cytokinin metabolism *in vivo* has to be elucidated.

## The purine (uptake) permease transporter family

A number of membrane protein families mediating intra- and intercellular transport of nucleobases are known to date. New findings expand their physiological role to include plant cytokinin and alkaloid metabolism as well as metabolism of vitamin B6.

Purine permease transporters (PUPs) belong to a family of small, highly hydrophobic membrane proteins. They are related to nucleotide-sugar transporters known from bacteria, archae, fungi and diverse eukaryotes within the drug metabolite transporter (DMT) super family (Jelesko, 2012). Homologs have been identified in several plant species like *Arabidopsis*, banana, rice, maize, tobacco and tomato (Gillissen et al., 2000; Hildreth et al., 2011; Goodstein et al., 2012; Jelesko, 2012). In *Arabidopsis* 21 PUP-like proteins are present with 9–10 transmembrane helices based on multiple prediction tools (Schwacke et al., 2003). The first member of the purine permease transporter family AtPUP1 was identified by complementation of a yeast mutant deficient in adenine uptake (*fcy2*) with an *Arabidopsis* cDNA expression library (Gillissen et al., 2000). To date, only AtPUP1, AtPUP2, AtPUP3, and NtNUP1, a tobacco PUP-like homolog, have been biochemically characterized. Direct uptake measurements demonstrated that AtPUP1 is able to transport adenine, *trans*-zeatin and pyridoxine (PN)—one of the three compounds that can be called vitamin B6 (Gillissen et al., 2000; Bürkle et al., 2003; Szydlowski et al., 2013). Moreover, competition studies and yeast complementation studies suggest additional transport abilities for kinetin, caffeine, cytosine, hypoxanthine, nicotine and the vitamin B6 forms pyridoxal (PL) and pyridoxamine (PM). The transport is supposed to work as a substrate-proton symport (Gillissen et al., 2000; Bürkle et al., 2003; Szydlowski et al., 2013). As well as AtPUP1, AtPUP2 mediated a proton-coupled adenine transport with the ability of *trans*-zeatin, *cis*-zeatin, kinetin, isopentenyladenine and benzylaminopurine transport (Bürkle et al., 2003). Moreover, direct uptake measurements with radiolabeled PN showed a weak import activity for AtPUP2 in yeast. In contrast, no substrate of AtPUP3 was identified so far (Bürkle et al., 2003). Uptake assays with radiolabeled substrates demonstrated that NtNUP1 from tobacco is able to transport nicotine but determination of kinetics weren't successful due to nicotine toxicity. However, kinetin atropine, anatabine, or anabasine added as competitors did not efficiently compete for nicotine uptake (Hildreth et al., 2011). Beyond, adenine is no substrate for NtNUP1 which indicates a high nicotine specificity (Hildreth et al., 2011). *Arabidopsis* seedlings expressing the AtPUP1-YFP fusion protein as well as transient expression studies of NtNUP1-GFP in tobacco mesophyll cells exhibited fluorescence at the plasma membrane, suggesting that NtNUP1 and AtPUP1 import apoplastic metabolites into the cell (Hildreth et al., 2011; Szydlowski et al., 2013). The subcellular localization of other PUP members remains unclear. In organ specific expression data for the AtPUP-like family from the Genevestigator database (Zimmermann et al., 2004), only a subset of 12 AtPUPs was represented (Cedzich et al., 2008). AtPUP1, AtPUP4, AtPUP11, AtPUP14, and AtPUP18 showed high expression levels throughout all tissues, whereas AtPUP2 and seven other members were only expressed at lower levels (Zimmermann et al., 2004; Cedzich et al., 2008). AtPUP1 is expressed in cotyledons, the stigma surface of siliques and epithem cells of hydatodes. In the epithem the transporter may play a role in retrieval of solutes from the xylem sap like purines, cytokinins or vitamin B6 (Bürkle et al., 2003; Hirose et al., 2008; Szydlowski et al., 2013). *Arabidopsis* cells possess a high affinity cytokinin transport system which shares properties with AtPUP1 and is compatible with the observed concentration range for cytokinins in xylem sap (Weiler and Ziegler, 1981; Komor et al., 1993; Beck and Wagner, 1994; Takei et al., 2001; Cedzich et al., 2008). However, the most prominent cytokinin form in xylem sap is tZ ribose, but also tZ is present and can be imported into the cells via AtPUP1 in general (Hirose et al., 2008). *AtPUP1* T-DNA mutants showed altered vitamin B6 composition of the guttation sap. The PN and PL contents were significantly increased by 253 and 64%, respectively compared to wildtype (Szydlowski et al., 2013). However, there is no information about alterations in purine or cytokinin content in those mutants, so the physiological relevance of AtPUP1 in purine and cytokinin recycling from the guttation sap remains unclear. Nevertheless, the scavenging of solutes from the xylem sap by AtPUP1 is an example of the plants effort to recycle valuable compounds and conserve energy. *AtPUP2* promoter expression was found in the vascular system of leaves and was limited to the phloem, indicating a potential role in phloem loading whereupon multiple transporters are required for long-distance transport. Other members of the AtPUP-like family are potential candidates to address this role (Bürkle et al., 2003; Jelesko, 2012). In *AtPUP3*-promotor-GUS plants, the staining was restricted to pollen, so AtPUP3 could mediate important transport processes of purines and cytokinins during pollen germination and tube elongation (Bürkle et al., 2003).

## Ureide permeases—transporters of heterocyclic nitrogen compounds

Ureide permeases (UPSs) form a protein superfamily of plant membrane transporters with five members in *Arabidopsis*, whereas three of them (AtUPS1, AtUPS2, and AtUPS5) have been characterized. The name originated from the ability of AtUPS1 to complement a yeast mutant defective in allantoin uptake (Desimone et al., 2002). Homologous proteins are present in legumes like french bean (PvUPS1) and soybean (GmUPS1-1 and GmUPS1-2), where ureides like allantoin and allantoic acid serve as long-distance transport molecules, representing up to 90% of the transported nitrogen (Desimone et al., 2002; Smith and Atkins, 2002). However, non-legumes like *Arabidopsis* use mainly amino acids, nitrate but also nucleosides and nucleobases like uracil in small amounts for long distance transport of nitrogen (Schmidt et al., 2004).

### Ureide permeases in legumes and their role in nucleotide metabolism and distribution

In tropical legumes like soybean (*Glycine max*) or french bean (*Phaseolus vulgaris*) ureides play a dominant role in primary nitrogen metabolism. After the initial fixation into glutamine, almost all fixed nitrogen is subsequently converted to the ureides allantoin and allantoic acid via inosine monophosphate (Smith and Atkins, 2002). This requires purine *de novo* synthesis, catabolic steps as well as transport processes to allow export from the root nodules to shoot tissues.

In soybean, two UPS homologs (GmUPS1-1 and GmUPS1-2) were characterized as high affinity allantoin transporters (Table 2). Soybeans with reduced *GmUPS1* transcript showed a decrease of ureides in roots and xylem sap, an accumulation of allantoin and allantoic acid in nodules and nitrogen deficiency symptoms in leaves (Collier and Tegeder, 2012). Moreover, the size of nodule cells and infected cells was smaller indicating that ureide transport activity in nodules is not only essential for nitrogen export and translocation to shoot but also for nodule development and function (Collier and Tegeder, 2012).

**Table 2:** **Nucleobase transporters of the PUP and UPS families**.

**Species (number of members)**	**Transporter (accession number)**	**Expression system, K_M_ [μM], substrate**	**Expression pattern**	**Physiological function**	**References**
**2.A.7.14 PURINE PERMEASE (PUP)**					
*Arabidopsis thaliana* (21 members)	AtPUP1 (At1g28230)	Y 30.0 (Adenine)	Constitutive except roots, high in leaf hydathodes, stigma surface	Reabsorbtion of nucleobases, cytokinine bases and Vitamine B6 from guttation fluid	Gillissen et al., 2000; Bürkle et al., 2003; Szydlowski et al., 2013
		Y 20.0 (Cytosine)			
		Y 40.0 (tZ)			
		Y 102.0 (PN)			
	AtPUP2 (At2g33750)	Y 22.6 (Adenine)	Vasculature of leaves	Uptake of nucleobases and cytokinine bases into phloem	Bürkle et al., 2003
	AtPUP3 (At1g28220)	N.A.	Pollen		
*Nicotiana tabacum*	NtPUP1 (G8A929)	Y (Nicotine)	Roots		Hildreth et al., 2011
*Oryza sativa* (12 members)	OsPUP7 (Os05g48300)	Y (Caffeine)	Vascular bundle system of culms, leaf sheaths, and roots	Transport of iP and iPR to other organs	Qi and Xiong, 2013
**2.A.7.19 UREIDE PERMEASE (UPS)**					
*Arabidopsis thaliana* (5 members)	AtUPS1 (At2g03590)	X 75 (Allantoin)	Young seedlings, Hypocotyl, roots		Desimone et al., 2002; Schmidt et al., 2004, 2006
		Y 52 (Allantoin)			
		X 5.9 (Uracil)			
		Y 24.0 (Xanthine)			
	AtUPS2 (At2g03530)	X 26 (Allantoin)	Seedlings (from day 4), hypocotyl, primary leaves	Utilization of substrates present in the rhizosphere	Desimone et al., 2002; Schmidt et al., 2004, 2006
		Y 75 (Allantoin)			
		X 6.2 (Uracil)			
		Y 7.0 (Xanthine)			
	AtUPS5 (At1g26440)	Y 35.6 (Allantoin)	Seedling roots, stem, leaves, flowers		Schmidt et al., 2006
		Y 38.5 (Uracil)			
		Y 6.8 (Xanthine)			
*Phaseolus vulgaris*	PvUPS1 (AY461734)	Y 98.0 (Allantoin)	Roots, source leaves, pods, seed coates	Delivery of allantoin to the vascular bundle and loading into the nodule phloem	Pélissier et al., 2004
*Glycine max*	GmUPS1-1 (Glyma01g07120)	Y 76.2 (Allantoin)	Nodule cortex, vascular endodermis	Export of allantoin and allantoic acid out of nodules	Collier and Tegeder, 2012
	GmUPS1-2 (Glyma02g12970)	Y 53.9 (Allantoin)	Nodule cortex, vascular endodermis	Export of allantoin and allantoic acid out of nodules	Collier and Tegeder, 2012

iP, isopentenyl adenine; iPR, isopentenyl adenine riboside; PN, pyridoxine; tZ, trans-zeatin; N.A., not analyzed; X, expressed in Xenopus oocytes; Y, expressed in yeast.

Also in french bean a gene (*PvUPS1*) encoding an allantoin transporter was identified and detected at the transcript level throughout the plant body. Expression in root tissues increased markedly upon nodulation indicating the importance of UPSs from legumes for delivery of ureides to the vasculature (Pélissier et al., 2004). After transport to sink tissues corresponding transporters are needed for cellular uptake. The biochemical characterization revealed similar transport properties for GmUPS1-1, GmUPS1-2, and PvUPS1. All three transporters show affinities for allantoin between 54 and 98 μM (Table 2). In addition, competition studies suggest that products of purine catabolism upstream of allantoin namely uric acid and xanthine might be transported. In contrast to PvUPS1, GmUPS1-1, and GmUPS1-2 mediated allantoin uptake is reduced to 50% by addition of allantoic acid, suggesting that this product of purine metabolism also acts as a substrate for soybean UPSs (Collier and Tegeder, 2012). Beyond, it was shown that uracil is a strong competitor for allantoin uptake mediated by GmUPS1-1 and GmUPS1-2. While allantoin and allantoic acid levels in legumes are high, the presence of uracil is marginal. For this reason, uracil is most probably no physiological relevant substrate for ureide permeases in legumes (Fujihara and Yamaguchi, 1978; Collier and Tegeder, 2012). Localization studies showed the presence of UPS transporters in the plasma membrane after heterologous expression in *Nicotiana benthamiana* leaves (Collier and Tegeder, 2012), in good agreement with the physiological function of these transporters.

### Ureide permeases in *Arabidopsis* and their role in nucleotide metabolism and distribution

Direct uptake measurements with AtUPS1, AtUPS2, and AtUPS5 indicated an allantoin transport activity, whereas an even higher affinity was observed for uracil (Table 2). As long distance transport of ureides does not seem to be of importance in non-legumes like *Arabidopsis* it can be hypothesized that the main function of AtUPS proteins is nucleobase transport (Desimone et al., 2002; Schmidt et al., 2004, 2006). Electrophysiological analyses with different AtUPS members synthesized in Xenopus oocytes and in yeast demonstrated a substrate-proton symport mode. Moreover, competition studies showed that AtUPS1 may also be able to use xanthine, uric acid, hydanthoin, cytosine, thymine, dihydrouracil and 5-FU as substrates (Desimone et al., 2002; Schmidt et al., 2004). AtUPS2 showed similar transport kinetics with only slight differences to AtUPS1 with respect to substrate specificity (Schmidt et al., 2004). AtUPS5 displays also a substrate spectrum quite similar to AtUPS1 and AtUPS2, but the substrate affinities for allantoin and uracil were lower. *AtUPS1* expression was increased during germination and early seed development followed by an upregulation of *AtUPS2* expression. The high expression of genes related to pyrimidine salvage in situations where the activity of enzymes for *de novo* synthesis are low indicates the dependency of cells on the salvage pathway and the distribution of their products (Stasolla et al., 2003; Schmidt et al., 2004). *AtUPS5* expression is high in epidermis, endodermis and cortex of roots and therefore could act partially redundant to AtUPS1 and AtUPS2 (Schmidt et al., 2006). In addition, data from *Arabidopsis* microarray experiments showed doubling of expression levels in senescent leaves (Winter et al., 2007) indicating a role in recycling of nitrogen-rich compounds under these conditions. A transport property required in all plants is related to catabolism of purines but is so far elusive. Here, allantoin generated in peroxisomes needs to be translocated to the endoplasmic reticulum (Werner and Witte, 2011) for which ureide permeases would be well suited. So far only GmUPS1-1 and 1-2 have been analyzed in this respect and localized to the plasma membrane (Collier and Tegeder, 2012). Corresponding analyses of UPS proteins from other species are missing so far.

## The nucleobase:cation symporter 1 family

The nucleobase:cation symporter 1 (NCS1) family consists of over 1000 proteins from bacteria, archaea, yeast, fungi and plants. Also known as purine-related transporters, they generally transport purines in a proton symport mode and consist of 12 transmembrane domains (TMs; Saier et al., 2009; www.tcdb.org). The uridine transporter FUI1 and the uracil transporter FUR4 from *Saccharomyces cerevisiae* (Chevallier and Lacroute, 1982; Zhang et al., 2006), the benzyl-hydantoin transporter MHP1 from *Microbacterium liquefaciens* (Weyand et al., 2008) and the cytosine- purine transporter FCYB from *Aspergillus nidulans* (Krypotou et al., 2012) are examples for well-studied NCS1 proteins.

In *Arabidopsis*, the plastidic nucleobase transporter PLUTO was identified as the sole NCS1 member with 23% sequence identity to FUR4. Other plants like maize (*Zea mays*), rice (*Oryza sativa*), wine (*Vitis vinifera*) and poplar (*Populus trichocarpa*) also possess a PLUTO homolog while *Brachypodium distachyon* even harbors two PLUTO homologs (Schwacke et al., 2003; Witz et al., 2014). Quite recently, a PLUTO homolog was also identified in *Clamydomonas reinhardtii* which is capable of transporting uracil, adenine, guanine, and allantoin suggesting that the solute specificity for plant NCS1 occurred early in plant evolution and is distinct from solute transport specificities of single cell fungal NCS1 proteins (Schein et al., 2013).

### Biochemistry and structure-function relations of PLUTO

A detailed biochemical characterization of *Arabidopsis* PLUTO was successful for the fulllength-protein of 599 amino acids after heterologous *PLUTO* expression in *E. coli* cells lacking the endogenous uracil transporter UraA. It was shown that PLUTO is capable of transporting uracil, guanine, and adenine with high affinities (Table 3; Witz et al., 2012). Furthermore, the addition of the protonophore CCCP inhibited uracil transport almost completely at low concentrations of 25 μM, suggesting that PLUTO functions as a nucleobase-proton symporter (Witz et al., 2012). The N-terminus of the protein contains a predicted target sequence for a localization in chloroplasts (Emanuelsson et al., 1999). After expression studies in *Arabidopsis* leaf protoplasts, a PLUTO-GFP fusion protein shows a localization in the inner envelope membrane of chloroplasts whereas a N-terminal truncated version does no longer reside in the envelope, but is mislocalized in the endomembrane system (Witz et al., 2012). In the EMBL database, PLUTO is annotated as a N-terminal truncated protein. Although this shorter version was not functional in the *E. coli* system and no longer targeted to plastids, it was shown to function in a yeast expression system (Mourad et al., 2012). So far, there are no data available that clearly proof the existence of a shorter PLUTO version in *Arabidopsis* and it is unclear whether both protein variants exist (Witz et al., 2014). However, PLUTO is expressed with a medium transcript level in all plant tissues and shows an increased expression in 2- to 10-day old seedlings, compared to 4-week old plants (Witz et al., 2012).

**Table 3:** **Nucleobase transporters of the NCS1 and NCS2 families**.

**Species (number of members)**	**Transporter (accession number)**	**Expression system, K_M_ [μM], substrate**	**Expression pattern**	**Physiological function**	**References**
**2.A.39 NUCLEOBASE:CATION SYMPORTER 1 FAMILY (NCS1)**					
*Arabidopsis thaliana* (1 member)	PLUTO (At5g03555)	B 0.38 (Adenine)	Nearly constitutively expressed, high in stem and seeds	Import of pyrimidine nucleobases into plastids	Witz et al., 2012, 2014
		B 6.29 (Guanine)			
		B 16.4 (Uracil)			
*Chlamydomonas reinhardtii*	CrNCS1 (A8J166)	Y 2.46 (Adenine)			Schein et al., 2013
		Y 5.90 (Uracil)			
		Y (Allantoin, Guanine)			
**2.A.40.1 NUCLEOBASE:CATION SYMPORTER 2 FAMILY (NCS2)/NUCLEOBASE ASCORBATE TRANSPORTER (NAT)**					
*Arabidopsis thaliana* (12 members)	AtNAT3 (At2g26510)	B 10.12 (Adenine)	Meristems and major leaf veins, central cylinder of roots, root tip		Maurino et al., 2006; Niopek-Witz et al., 2014
		B 4.85 (Guanine)			
		B 19.95 (Uracil)			
	AtNAT12 (At2g27810)	B 1.74 (Adenine)	Constitutively high throughout plant development		Maurino et al., 2006; Niopek-Witz et al., 2014
		B 2.44 (Guanine)			
		B 29.83 (Uracil)			
*Zea mays*	LPE1 (GBWI-87088)	F 30.0 (Xanthine)	N.A.		Argyrou et al., 2001
**2.A.40.7 NUCLEOBASE:CATION SYMPORTER 2 FAMILY (NCS2)/AZGA-LIKE PROTEIN FAMILY (AZG)**					
*Arabidopsis thaliana* (2 members)	AtAzg1 (At3g10960)	Y (Adenine)	Constitutively expressed^*^	T-DNA insertion mutant reveals a marked resistance to growth in the presence of 8-azaadenine and 8-azaguanine	Mansfield et al., 2009
		Y (Guanine)			
	AtAzg2 (At5g50300)	Y (Adenine)	Roots, pollen and developing seed^*^	T-DNA insertion mutant reveals a marked resistance to growth in the presence of 8-azaadenine and 8-azaguanine	Mansfield et al., 2009
		Y (Guanine)			

Y, expressed in yeast; B, expressed in E. coli; F expressed in fungi; N.A., not analyzed;

* Data taken from Genevestigator (Zimmermann et al., 2004).

First structural information about NCS1 proteins emerged with the crystal structure of the benzyl-hydantoin transporter MHP1 from *Microbacterium liquefaciens* (Weyand et al., 2008). This protein consists of 12 transmembrane segments arranged in two repeating halves (TM1–TM5 and TM6–TM10, followed by TM11 and TM12) and forming a cavity for substrate binding in the center of the protein. Furthermore, Weyand et al. (2008) identified 34 highly conserved amino acids among NCS1 proteins from bacteria and fungi. In PLUTO, 30 of these residues are also conserved (Witz et al., 2012). This is of high interest especially as there was not much known about the substrate binding site of NCS1 members with other substrates like purines and pyrimidines, e.g., uracil as it is the case for PLUTO.

**Figure 1:**
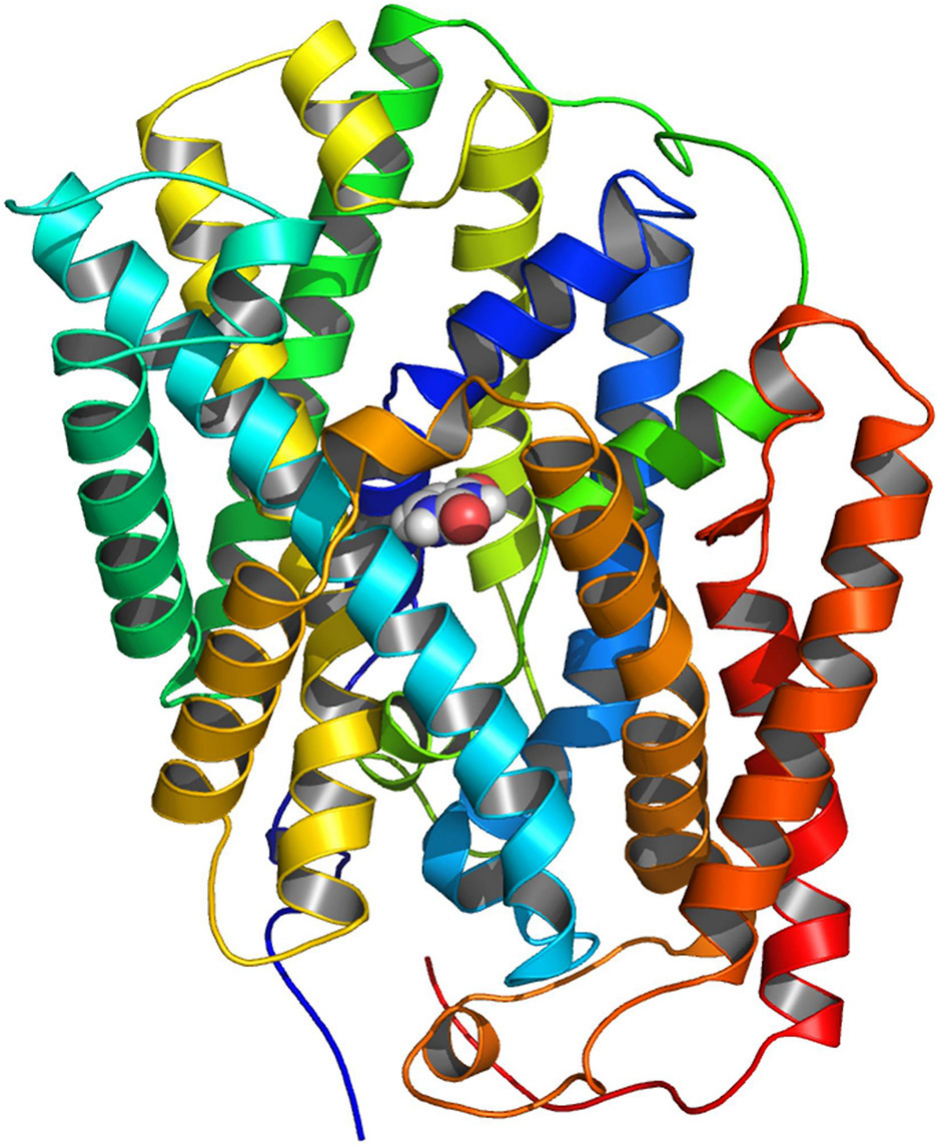
**Homology model of PLUTO, the plastidic nucleobase transporter from *Arabidopsis* with a uracil molecule located to the identified substrate binding site**. Homology modeling was performed based on the outward open conformation of MHP1, the benzyl-hydanthoin transporter from *Microbacterium liquefaciens*. Reproduced from the model presented in Witz et al. (2014).

Quite recently, a homology model of PLUTO based on the crystal structure of the benzyl hydantoin transporter MHP1 from *Microbacterium liquefaciens* was built and the structural stability was supported by molecular dynamics simulations (Witz et al., 2014; Figure 1). The model is composed of 12 TM helices, in which 10 TMs form a compact core followed by two additional TMs. The structure can be divided into two topologically distinct subdomains. The first is a four helix bundle comprising TM1 and TM2 and their pseudo two-fold equivalents TM6 and TM7 and is proposed to form the substrate binding site. The second subdomain is another motif of four helices formed by TM3 and TM4 and their pseudo two-fold equivalents, TM8 and TM9 which may shape an ion-binding site (Figure 1). Polar side chains of residues Glu-227 and backbones of Val-145, Gly-147, and Thr-425 with respect to PLUTO are proposed to form the binding site for the three substrates uracil, adenine, and guanine (Witz et al., 2014). The analysis of several PLUTO mutants after expression in *E. coli* cells lacking the endogenous uracil transporter UraA identified Glu-227 as an important residue for uracil and to a less extent also for guanine transport. PLUTO residue Thr-425 in addition to Gly-147 was identified as an adenine interaction partner whereas residues Glu-227, Val-145, Gly-147, and Thr-425 are restricted to guanine transport. Furthermore, substrate competition studies as well as docking studies clearly demonstrated that uracil and guanine exhibit a similar binding mode whereas adenine binds deeper into the catalytic pocket of PLUTO (Witz et al., 2014). In addition, competition studies with purine related compounds identified hypoxanthine as an additional putative substrate of PLUTO as it shows marked inhibition of guanine transport (Witz et al., 2014).

### PLUTO and its role in nucleotide metabolism

It has recently been shown that the pyrimidine *de novo* synthesis is finalized in the cytosol. Subsequently, pyrimidines must be imported into plastids in form of precursors to allow the synthesis of nucleic acids in this compartment (Witz et al., 2012). The salvage of nucleosides and nucleobases represents an alternative to the *de novo* synthesis and is less energy consuming (Möhlmann et al., 2010). Also in plastids, several salvage enzymes occur. These are for example the plastidic enzyme uracil phosphoribosyl transferase (UPP) accepting uracil as a substrate and leading to the formation of uridine-5′-monophosphate (UMP). Mutants lacking this enzyme showed severe growth defects and chlorosis (Mainguet et al., 2009). In addition, two plastidic uridine kinases namely UKL1 and UKL2 have been characterized in great detail and a double mutant defective in *UKL1* and *UKL2* genes had severe developmental defects and reduced biomass accumulation indicating the importance of salvage reactions in plastids (Chen and Thelen, 2011). Until now, nothing is known about the source of uridine as a substrate for plastidic uridine kinases. At least, a corresponding transporter at the plastid envelope has not been identified so far. Furthermore, plastids have to import uracil and thymine for the catabolism of pyrimidines. Hereby, uracil appears as a breakdown product from cytosolic nucleoside hydrolase 1 (NSH1; Jung et al., 2009) and has to be imported into plastids as the first enzyme of a three-step catabolism, the pyrimidine degradation enzyme 1 (PYD1) is also located in the plastid stroma (Zrenner et al., 2009; Cornelius et al., 2011). So far, PLUTO represents the only characterized transport protein in *Arabidopsis* mediating the import of uracil as a precursor for the synthesis of nucleotides via salvage reactions in plastids. The high expression of PLUTO in 2–10 day old seedlings supports its role in pyrimidine metabolism because in this developmental stage, the expression of NSH1 and PYD1 is also increased (Witz et al., 2012).

## The nucleobase:cation symporter 2 family/nucleobase ascorbate transporter family

The nucleobase-ascorbate transporter (NAT) family, belonging to the nucleobase:cation symporter 2 (NCS2) family, represents the largest and most conserved class of nucleobase transporters (De Koning and Diallinas, 2000). It includes more than 2000 putative members in all major taxa of organisms like eubacteria, archea, filamentous fungi, plants, insects, nematodes, mammals and humans (De Koning and Diallinas, 2000; Frillingos, 2012). Remarkably, most protozoa and *Saccharomyces cerevisiae* do not possess NAT proteins and only a few NAT proteins have been biochemically characterized so far (Frillingos, 2012). Transporters of known function are for example UapA and UapC from *Aspergillus nidulans* (Diallinas et al., 1995), UraA from *Escherichia coli* (Andersen et al., 1995; Lu et al., 2011), PyrP from *Bacillus subtilis* (Turner et al., 1994), Lpe1 from *Zea mays* (Argyrou et al., 2001) or the mammalian transporters rSNBT (Yamamoto et al., 2009), SVCT1 and SVCT2 (Tsukaguchi et al., 1999). These transporters are very specific for the cellular uptake of either uracil, xanthine or uric acid (bacteria, fungi, plants) or ascorbate (mammalians; Frillingos, 2012). NAT proteins usually consist of 400–650 amino acids and 12–14 transmembrane segments. Moreover, a common feature is the existence of the NAT-signature upstream from transmembrane segment 9 (Koukaki et al., 2005) and a QH-motif in transmembrane segment 1 (Pantazopoulou and Diallinas, 2007). Both motifs are highly conserved among NAT proteins and necessary for a proper function of UapA, UapC, or YgfO from *Aspergillus nidulans* (Gournas et al., 2008).

In *Arabidopsis* 12 members of the NAT family, AtNAT1-12, have been identified and analyzed concerning their expression patterns during growth and development (Maurino et al., 2006). Based on a multiple sequence alignment, the AtNAT proteins split into five clades which correlates with their expression during the life cycle of *Arabidopsis* (Maurino et al., 2006). Some of the members of this gene family show ubiquitous expression (e.g., AtNAT12), while the expression of other AtNAT genes is restricted to specific tissues (AtNAT7, AtNAT8, AtNAT9; Maurino et al., 2006; Table 3). In addition, most AtNATs show pronounced expression is vascular tissues, indicating that these proteins might have a function in long-distance transport of metabolites (Maurino et al., 2006). All analyzed mutant lines of AtNAT family members including several double- and triple mutants belonging to the same clade lack obvious phenotypical differences compared to the wild type. These observations might be due to a high redundancy of NAT functions in plants. Either the functions can be compensated by other NAT genes or by other nucleobase transporters (Maurino et al., 2006). Transient expression studies of AtNAT-GFP fusion constructs in different systems showed that AtNAT7, AtNAT8 and also AtNAT12 are located to the plasma membrane (Maurino et al., 2006), although AtNAT12 possesses a hydrophilic N-terminus with a prediction for a localization in the chloroplast (Schwacke et al., 2003).

The biochemical characterization of these proteins was not successful by the complementation of *Aspergillus nidulans* strains lacking functional NATs, heterologous expression studies in yeast, oocytes or measurements of radiolabeled substrates in whole plants (Maurino et al., 2006). Quite recently, the heterologous expression of *AtNAT3* and *AtNAT12* in *E. coli* cells lacking the endogenous uracil transporter UraA allowed for a detailed biochemical characterization of these two NAT proteins showing that they mediate high affinity uptake of uracil, adenine and guanine (Niopek-Witz et al., 2014).

## The nucleobase:cation symporter 2 family/the AzgA-like protein family

The AzgA proteins define a group of membrane proteins which may be distantly related to the NAT family and homologs of unknown function are found in plants, fungi, bacteria and Archaea. In *Aspergillus nidulans*, AzgA has been identified as a proton-symporter specific for hypoxanthine, guanine, and adenine (Cecchetto et al., 2004). Two proteins with significant similarity to the AzgA adenine-guanine-hypoxanthine transporter of *Aspergillus nidulans*, namely AtAzg1 and AtAzg2, have also been identified in *Arabidopsis*. These proteins share 36.5 and 38.5% identical amino acids with AzgA, respectively (Mansfield et al., 2009). Homozygous mutant lines of the allele *AtAzg1-1* and *AtAzg1-2* showed increased resistance on 8-azaguanine and 8-azaadenine compared to the wildtype and this effect was even more pronounced in *AtAzg1* and *AtAzg2* double mutants. Growth tests of *S. cerevisiae* cells expressing *AtAzg1* and *AtAzg2* on medium supplemented with the toxic analogs 8-azaadenine or 8-azaguanine as well as direct uptake studies with radiolabeled [^3^H]-adenine and [^3^H]-guanine indicated that AtAzg1 and AtAzg2 are capable of transporting adenine and guanine (Mansfield et al., 2009). Both proteins are integral membrane proteins with 10 transmembrane segments and a prediction for a localization in chloroplasts (Schwacke et al., 2003). However, there is no experimental evidence for a localization of AtAzg proteins in the chloroplast envelope. Mansfield et al. (2009) suggested that AtAzg1 is located to the plasma membrane necessary for adenine and guanine import while AtAzg2 might be located to the plastidic envelope based on the predicted targeting motif. However, several prediction tools reveal a chloroplastidic localization to be more likely for AtAzg1 rather than for AtAzg2 (Schwacke et al., 2003).

## Conclusion

In the last years it became apparent that nucleoside and nucleobase transporters accept a diverse range of substrates, far more than judged by the given name of these proteins. Consequently, the adjudicated physiological functions also increased. To briefly summarize, ENTs represent the sole family of nucleoside transporters in plants, mediating transport of purine and pyrimidine nucleosides across the plasma membrane and the tonoplast. Within the large number of nucleobase transport families, PUPs exhibit a broad substrate spectrum including purine nucleobases, cytokinins, vitamins and alkaloids. UPS proteins play a major role in ureide long distance transport in tropical legumes whereas in non-legumes pyrimidine nucleobase transport predominates. From the NCS1 protein family only one member is present in higher plants with PLUTO, the plastidic nucleobase transporter. In contrast, NCS2 members reside at the plasma membrane where they catalyze transport of purine and pyrimidine nucleobases. It can be anticipated that research in the field of nucleoside and nucleobase transport will continue to develop fruitfully in future, implementing aspects from crop plants.

### Conflict of interest statement

The Guest Associate Editor Ilka Haferkamp declares that, despite being affiliated to the same institution as the authors, the review process was handled objectively and no conflict of interest exists. The authors declare that the research was conducted in the absence of any commercial or financial relationships that could be construed as a potential conflict of interest.
